# Extracorporeal shockwave therapy rescued mouse critical limb ischemia via upregulating GPR120 against inflammation and promoting angiogenesis for restoring the blood flow in ischemic zone-experimental study

**DOI:** 10.1097/JS9.0000000000002243

**Published:** 2025-01-28

**Authors:** Pei-Hsun Sung, Jui-Ning Yeh, Tsung-Cheng Yin, Han-Tan Chai, John Y. Chiang, Chi-Ruei Huang, Yi-Ling Chen, Mel S. Lee, Hon-Kan Yip

**Affiliations:** a Division of Cardiology, Department of Internal Medicine, Kaohsiung Chang Gung Memorial Hospital and Chang Gung University College of Medicine, Kaohsiung, Taiwan; b Institute for Translational Research in Biomedicine, Kaohsiung Chang Gung Memorial Hospital, Kaohsiung, Taiwan; c Center for Shockwave Medicine and Tissue Engineering, Kaohsiung Chang Gung Memorial Hospital, Kaohsiung, Taiwan; d Institute of Nephrology and Blood Purification, the First Affiliated Hospital of Jinan University, Jinan University, Guangzhou, China; e Department of Cardiology, The First Affiliated Hospital, Jinan University, Guangzhou, China; f Department of Orthopaedic Surgery, Kaohsiung Chang Gung Memorial Hospital and Chang Gung University College of Medicine, Kaohsiung, Taiwan; g Department of Computer Science and Engineering, National Sun Yat-Sen University, Kaohsiung, Taiwan; h Pao-Chien Hospital, Pingtung, Taiwan; i School of Medicine, College of Medicine, Chang Gung University, Taoyuan, Taiwan; j Department of Medical Research, China Medical University Hospital, China Medical University, Taichung, Taiwan

**Keywords:** angiogenesis, critical limb ischemia, extracorporeal shock wave, G protein-coupled receptor 120, inflammation

## Abstract

**Background:**

This study tested the hypothesis that extracorporeal shockwave therapy (ECSWT) effectively rescues critical limb ischemia (CLI) in mice through the upregulation of GPR120, which protects against inflammation and angiogenesis to restore blood flow in the ischemic area.

**Methods and results:**

Compared with the control, ECSWT-induced GPR120-mediated anti-inflammatory effects significantly suppressed the expression of inflammatory signaling biomarkers (TAK1/MAPK family/NF-κB/IL-1β/IL-6/TNF-α/MCP-1) in HUVECs, and these effects were abolished by silencing GPR120 or by the GPR120 antagonist AH7614 (all *P* < 0.001). C57BL/6 mice (*n* = 40) were equally categorized into Groups 1 (sham-operated control), 2 (CLI), 3 (CLI + ECSWT), and 4 (CLI + ECSWT + AH7614). By Days 7, 14, and 28 just prior to harvesting the quadriceps muscle, the laser Doppler results showed that the ratio of ischemia to normal blood flow (INBF) in the CLI area was highest in Group 1, lowest in Group 2, and significantly greater in Group 3 than in Group 4 (all *P* < 0.0001). Endothelial cell markers (CD31/vWF) and GPR120 + cells exhibited identical patterns of INBF among the groups, whereas angiogenesis biomarkers (CXCR4/SDF-1/VEGF/VEGFR2) were significantly and progressively upregulated from Groups 1 to 4 (all *P* < 0.0001). The protein levels of inflammation (MMP-9, IL-6, and TNF-α) and oxidative stress (NOX-1 and NOX-2) and the cellular levels of inflammation (CD68+)/DNA damage (γ-H2AX+) displayed opposite patterns, whereas the small vessel density in the CLI area displayed an identical pattern of INBF among the groups (all *P* < 0.0001).

**Conclusions:**

ECSWT rescued CLI by increasing GPR120-mediated suppression of inflammation and enhancing angiogenesis via activation of VEGFR2.

## Graphical abstract

**Figure:**
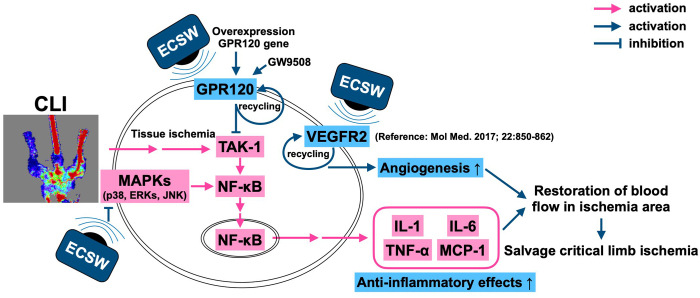

## Introduction

Peripheral artery disease (PAD) is one of the most common pathological conditions of atherosclerosis. Clinical studies have demonstrated that patients with severe limb ischemia account for up to 20%–40% of combined amputations and mortality^[1]^. The underlying pathogenesis is very complex and includes imbalanced lipid accumulation and chronic inflammatory cell infiltration in the vascular wall^[2,3]^. Gremmels *et al* reported that the inflammatory biomarkers interleukin (IL)-6, IL-8, and IL-10 were markedly elevated in patients with primary outcomes of major amputation and death^[4]^. On the other hand, anti-inflammatory therapy with IL-1 antagonists has been reported to have a significantly lower rate of recurrent cardiovascular events^[5]^. These issues prompted the investigation of a new and safe therapeutic modality for reducing the incidence of PAD.

Extracorporeal shock wave therapy (ECSWT) is a noninvasive mechanical treatment that has been found to be safe and effective for treating several musculoskeletal disorders because of its anti-inflammatory properties^[6]^. However, the underlying mechanism by which ECSWT protects against inflammation remains unclear. Interestingly, in recent decades, several biological effects induced by ECSWT, including enhancements in wound healing, angiogenesis, and the inhibition of painful stimuli and inflammatory responses, have been well recognized by investigators^[7]^. In addition, many studies using animal models have attempted to extend ECSWT to different disease entities, including myocardial infarction, critical limb ischemia (CLI), diabetic neuropathy, carotid artery stenosis, and interstitial cystitis^[8–12]^, with promising results. Basic research has further demonstrated that ECSWT can trigger the ligand-independent vascular endothelial growth factor receptor 2 (VEGFR2) signaling pathway to increase angiogenesis through the formation of mechanosensory complexes and receptor recycling^[13,14]^. In addition to VEGFR2, we assume that other receptors may be driven by ECSWT in endothelial cells.

Although clear signal transduction of ECSWT-induced angiogenesis has been demonstrated^[8–14]^, the exact anti-inflammatory mechanism remains poorly understood. *Protein*-coupled receptor 120 (GPR120), a receptor from the G protein-coupled receptor (GPCR) family, is activated by fatty acids and mediates many cellular processes^[15,16]^. In addition, studies have clearly demonstrated that GPR120 is expressed in multiple cell types and is associated with various physiological processes, including improvements in islet function, inflammation, and adipogenesis^[17–20]^. GPR120 activation by omega-3 fatty acids (ω-3 FAs) triggers the coupling of β-arrestin-2 and the release of transforming growth factor (TGF)-β active kinase 1 (TAK1), which inactivates TAK1 and upregulates inflammatory cascades associated with the signaling processes of NF-κB and mitogen-activated protective kinases (MAPKs)^[15,21]^. The discovery of an anti-inflammatory reaction mediated by GPR120 suggests the therapeutic potential of this receptor against inflammatory diseases^[22]^. Interestingly, Wu *et al* reported that the activation of GPR120 signaling in human colorectal carcinoma promotes angiogenesis through the induction of vascular endothelial growth factor (VEGF), which is an important cytokine that enhances angiogenesis^[23,24]^. Our previous study revealed that ECSWT abolished inflammation and restored urothelial barrier integrity in the context of interstitial cystitis through the induction of the fatty acid receptor GPR120 (i.e., a sensing receptor for the ECSWT)-associated signaling pathway in urothelial cells^[25]^. Accordingly, we propose that both VEGFR2 and GPR120 may be correlated during the progression of tissue/organ repair and regeneration. Taken together, the present study aimed to investigate the impact of ECSWT on alleviating CLI through the activation of GPR120-mediated anti-inflammatory responses and VEGFR2-mediated angiogenesis in mice.

## Materials and methods

### Ethical issues

All animal experimental protocols and procedures were approved by the Institute of Animal Care and Use Committee at our hospital. Animals were housed in an AAALAC-approved animal facility in our hospital (IACUC protocol no. 2021032303). This work has been reported in accordance with the ARRIVE guidelines (Animals in Research: Reporting In Vivo Experiments)^[26]^.

### Cell culture

Human umbilical vein endothelial cells (HUVECs) were purchased from BCRC (Taiwan), and the culture medium was purchased from ScienCell. The HUVECs were maintained in culture medium and grown at 37°C in a humidified atmosphere of 5% CO_2_. For the experiments, confluent cells in a cell culture dish were trypsinized and seeded into a culture flask (25T) at a density of 3 × 10^5^ cells/25T. At 80%–90% confluency, the cells were collected for the specific study.

### Small interfering RNA (siRNA) silencing of GPR120

siRNA for mouse GPR120 (Thermo Fisher Scientific) and scrambled siRNA were obtained from Thermo Fisher Scientific. The HUVECs (1 × 10^5^ cells per well in a six-well plate) were transfected according to the manufacturer’s instructions. After transfection, total RNA and protein were extracted from the cells to quantify the relative expression level of GPR120 by RT-PCR and immunoblotting.

### Transfection of cells with GPR120 protein expression vector

GPR120 protein expression vector pcDNA3.1-hygro (+) was purchased from Genomics Inc. (New Taipei City, Taiwan). The expression vector was amplified by *E. coli* (DH5a strain). Transfection of cells with plasmids was performed with Lipofectamine 3000 (Invitrogen, Life technologies, Carlsbad, CA, USA) according to the manufacturer’s instructions but with slight modifications. Cells were replated 24 h before transfection at a density of 1 × 10^6^ cells in 10 mL of fresh culture medium in a 10-cm plastic dish. For transfection, 30 μL of Lipofectamine 3000 was incubated with 15 μg of indicated expression vector at room temperature. The complex was incubated with cells at 37°C in a humidified atmosphere of 5% CO_2_ before being harvested.

### Procedure and protocol for ECSW application to petri dish cells

The procedure used for the application of ECSWs to culture cells was reported in our previous study.^14^ In detail, the HUVECs were initially cultured in a cell culture dish (iBidi GmbH, Martinsried, Germany). After the culture cells reached full confluence, the petri dish was carefully removed from the culture box. To avoid contamination, the culture disk was placed into an aseptic culture flask, after which ECSWs were applied to the HUVECs (refer to Supplementary Figure 1, http://links.lww.com/JS9/D772).

### Enzyme-linked immunosorbent assay (ELISA)

The levels of IL-1β and IL-6 in the serum were determined using commercially available ELISA kits according to the manufacturer’s instructions.

### Animals utilized for the study

Pathogen-free, 7–12-week-old adult male C57BL/6 mice (Charles River Technology, BioLASCO Taiwan Co., Ltd., Taiwan) were randomly allocated to four experimental groups after a 7-day adaptation period in this study.

### Mouse CLI model

Adult male mice were anesthetized preoperatively with 3% isoflurane prior to CLI induction. Under sterile conditions, the left femoral artery, small arterioles, and circumferential femoral artery were exposed and ligated over their proximal and distal portions prior to removal.

### Animal grouping and treatment strategy

Adult male C57BL/6 mice (*n* = 40) were equally categorized into Group 1 (sham-operated control [SC]), Group 2 (CLI with 1.5 mL N/S injection into ischemic quadriceps muscles 24 h after the CLI procedure), Group 3 (CLI + ECSWT [0.15 mJ/mm^2^ with 180 impulses] applied to the CLI area at Days 1, 3, 5, and 7 after CLI induction), and Group 4 (CLI + ECSWT [0.15 mJ/mm^2^ with 180 impulses] + AH7614 [0.5 mg/kg; a GPR120 antagonist]) at Days 1, 3, 5, and 7 after CLI induction, respectively. The animals were euthanized, and the quadriceps muscles were harvested on Day 28 after the CLI procedure. The dose of AH7614 utilized in the present study was based on a previous report^[27]^.

Application of ECSW therapy to the CLI area on Days 1, 3, 5, and 7 after CLI induction (Supplementary Figure 1, http://links.lww.com/JS9/D772)

The animals in Groups 3 and 4 were anesthetized with 3% isoflurane prior to applying ECSW therapy for CLI induction. Under sterile conditions, the ECSW probe (Duolith SD1; STORZ MEDICAL company) was adjusted to a position that was in close contact with the CLI area. After the probe position was well fixed, WCSW energy (0.15 mJ/mm^2^ with 180 impulses) was applied to the CLI area (refer to Supplementary Figure 1, http://links.lww.com/JS9/D772).

### Measurement of the ratio of ischemia to normal blood flow (INBF) in the CLI area with laser Doppler

The animals in each group were anesthetized by inhalation of isoflurane (2.0%) prior to CLI induction and on Days 1, 7, 14, and 28 after CLI induction for laser Doppler study. Each mouse was placed supine on a warming pad (37°C), and blood flow was detected in both inguinal areas using a laser Doppler scanner (MoorLDLS, Moor Instruments, UK). The ratio of blood flow in the left (ischemic) and right (normal) legs (i.e., the INBF) was carefully evaluated, collected, and then entered into the database computer. On Day 28, all the animals were euthanized, and the quadriceps muscles were isolated and collected for individual study.

### Immunofluorescence staining

Frozen sections were incubated with primary antibodies specific for CD68 (1:500, Abcam), γ-H2AX (1:750, Abcam), cytochrome C (1:500, Santa Cruz), Hsp60 (1:1000, Abcam), alpha-smooth muscle actin (α-SMA) (1:700, Sigma‒Aldrich), CD31 (1:200, Merck Millipore) and G protein-coupled receptor 120 (GPR120) (1:300, Abcam) at 4°C overnight. Three sections of samples dissected from the quadriceps muscles were analyzed for each animal. For quantification, six randomly selected high-power fields (HPFs) were analyzed in each section.

### Immunoblotting of quadriceps muscles harvested from the LCI area

Frozen tissues of harvested quadriceps muscles from the CLI area or cell samples were mechanically homogenized using 1× RIPA buffer (Cell Signaling Technology) containing 1× protease inhibitor cocktail (Roche). Protein lysates were separated by SDS-PAGE on 8%‒12% acrylamide gradients, transferred to a nitrocellulose membrane (Bio-Rad Laboratories) or polyvinylidene difluoride membrane (Sigma‒Aldrich) and probed with specific monoclonal antibodies. Immunoreactive bands were detected by enhanced chemiluminescence (EMD Millipore) and quantified with Quantity One Image Software (Bio-Rad Laboratories). The membranes were incubated with the indicated primary antibodies (phosphorylated (p)-TAK1 [1:1000, Cell Signaling Technology], p-nuclear factor [NF]-κB [1:1000, Cell Signaling Technology], monocyte chemoattractant protein [MCP]-1 [1:1000, Abcam], IL-6 [1:1000, Abcam], IL-1β [1:1000, Cell Signaling Technology], tumor necrosis factor [TNF]-alpha [1:1000, Cell Signaling Technology], GPR120 [1:2000, Santa Cruz], NOX-1 [1:2000, Sigma‒Aldrich], NOX-2 [1:1000, Sigma‒Aldrich], von Willebrand factor [vWF] [1:2000, Abcam], CD31 [1:3000, Abcam], CXCR4 [1:1000, Abcam], stromal cell-derived factor [SDF]-1α [1:1000, Cell Signaling Technology], VEGF [1:1000, Abcam], VEGF receptor [VEGFR] 2 [1:1000, Abcam], matrix metalloproteinase 9 [MMP-9] [1:2000, Abcam], dynamin-related protein 1 [DRP1] [1:1000, Cell Signaling Technology], cleaved (c)-caspase3 [1:1000, Cell Signaling Technology], c-PARP [1:1000, Cell Signaling Technology], Bax [1:2000, Abcam], γ-H2AX [1:1000, Cell Signaling Technology], p-JNK [1:2000, Abcam], p-ERK1/2 [1:5000, EMD Millipore], p-P38 [1:3000, Sigma–Aldrich], and β-actin [1:6000, EMD Millipore]). Horseradish peroxidase-conjugated anti-mouse immunoglobulin IgG (1:6000, Sigma‒Aldrich) and horseradish peroxidase-conjugated anti-rabbit immunoglobulin IgG (1:6000, Sigma‒Aldrich) were used as secondary antibodies.

### Small vessel staining and definition of small vessels in the CLI area

The procedure and protocol were based on our previous report^[28]^. Immunohistochemical (IHC) staining of small blood vessels was performed with α-SMA (1:400) as the primary antibody at room temperature for 1 h, followed by washing with PBS three times. Ten minutes after the addition of anti-mouse-HRP-conjugated secondary antibody, the tissue sections were washed with PBS three times. Then, 3,3′-diaminobenzidine (0.7 gm/tablet) (Sigma) was added, followed by washing with PBS three times after 1 min. Finally, hematoxylin was added as a counterstain for the nuclei, followed by washing twice with PBS after 1 min. Three quadriceps sections from each mouse were analyzed. For quantification, three randomly selected HPFs (400×) were analyzed in each section. The mean number per HPF for each animal was then determined by summing all the numbers divided by 9. A small vessel was defined as a vessel with a diameter ≤25.0 μM)

### Statistical analysis

All values are expressed as the means ± SDs. In accordance with the normality of the data, the independent *t*-test or Mann–Whitney *U* test was applied to analyze differences between two groups, and differences among more than three independent groups were analyzed via ANOVA or Kruskal–Wallis analysis. Statistical analysis was performed using SPSS statistical software for Windows, version 13 (SPSS for Windows, version 13; SPSS, Chicago, IL, USA). A value of *P* less than 0.05 was considered statistically significant.

## Results

### Pilot studies for evaluation of ECSWT increased GPR120 protein expression in a dose-dependent and time-dependent manner

**Figure 1.**
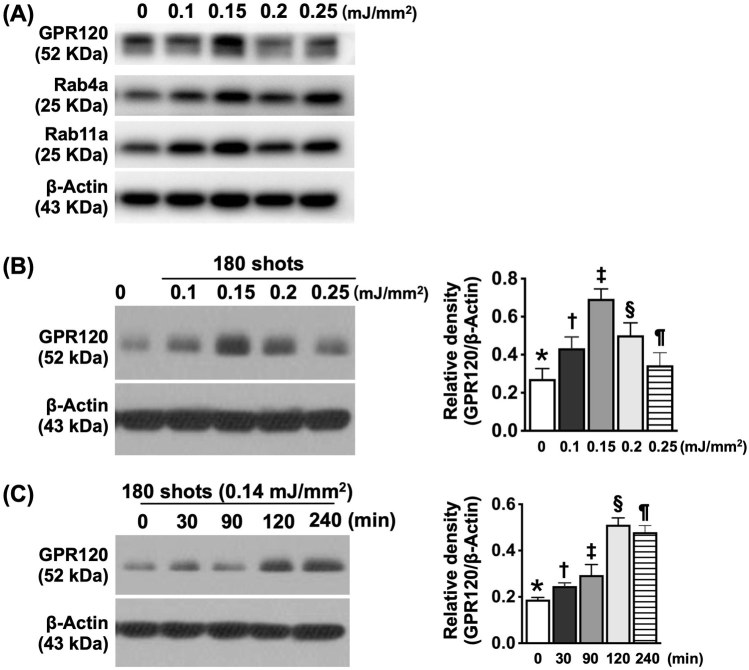
Pilot studies for evaluation of ECSWT increased GPR120 protein expression was in a dose-dependent and a time-dependent manner. (A) A pilot study of stepwise increased ECSW energy (from 0.10, 0.15, 0.20, and 0.25 mJ/mm^2^) on HUVECs for elucidating the optimal ECSW energy (*n* = 1). The results showed that lower energy (0.15 mJ/mm^2^) of ECSW was the maximal energy effect for upregulation of maximal GPR120 protein expression. On the other hand, higher energy (0.25 mJ/mm^2^) would attenuate the protein expression of GPR120 at the time point of 120 min after ECSWT. Another two G protein-coupled receptors, including Rab4a and Rab11a, also expressed a similar effect of GPR120 after ECSWT. (B) Analytical result of GPR120 protein expression at stepwise increased ECSW energy, * vs. other groups with different symbols (†, ‡, §, ¶), *P* < 0.001, *P* for trend <0.01. Note that * indicated the control group, i.e., the ECSW energy = 0 (*N* = 3 for each group). (C) When application of ECSW energy 0.14 mJ/mm^2^ and 180 impulses to the HUVECs and collection of the cells at time points of 30, 90, 120, and 240 min after shock wave treatment for Western blot analysis, we found that the maximal protein expression of GPR120 was identified at the time point of 120 min. analytical result of the protein expression of GPR120, * vs. other groups with different symbols (†, ‡, §, ¶), *P* < 0.001, *P* for trend <0.01. Note that * indicated the control group, i.e., the time point of 0 min. All statistical analyses were performed by one-way ANOVA, followed by Bonferroni multiple comparison post hoc test (*N* = 3–5 for each group). Symbols (*, †, ‡, §, ¶) indicate significance (at 0.05 level). ECSWT, extracorporeal shock wave therapy; HUVECs, human umbilical vein endothelial cells.

First, to determine the level of GPR120 protein expression in response to different energy levels of ECSWT, we applied four different low-energy levels (i.e., stepwise increases in ECSWT energy from 0.10, 0.15, 0.20, and 0.25 mJ/mm^2^ and a total of 180 shots) to HUVECs. The protein expression of GPR120 was determined by immunoblotting and is displayed in Fig. 1A. The results revealed that a lower energy (0.15 mJ/mm^2^) of ECSWT was the optimal energy effect for the upregulation of maximal GPR120 protein expression. On the other hand, higher energy (0.25 mJ/mm^2^) attenuated the protein expression of GPR120 at 120 min after ECSWT.

**Figure 2.**
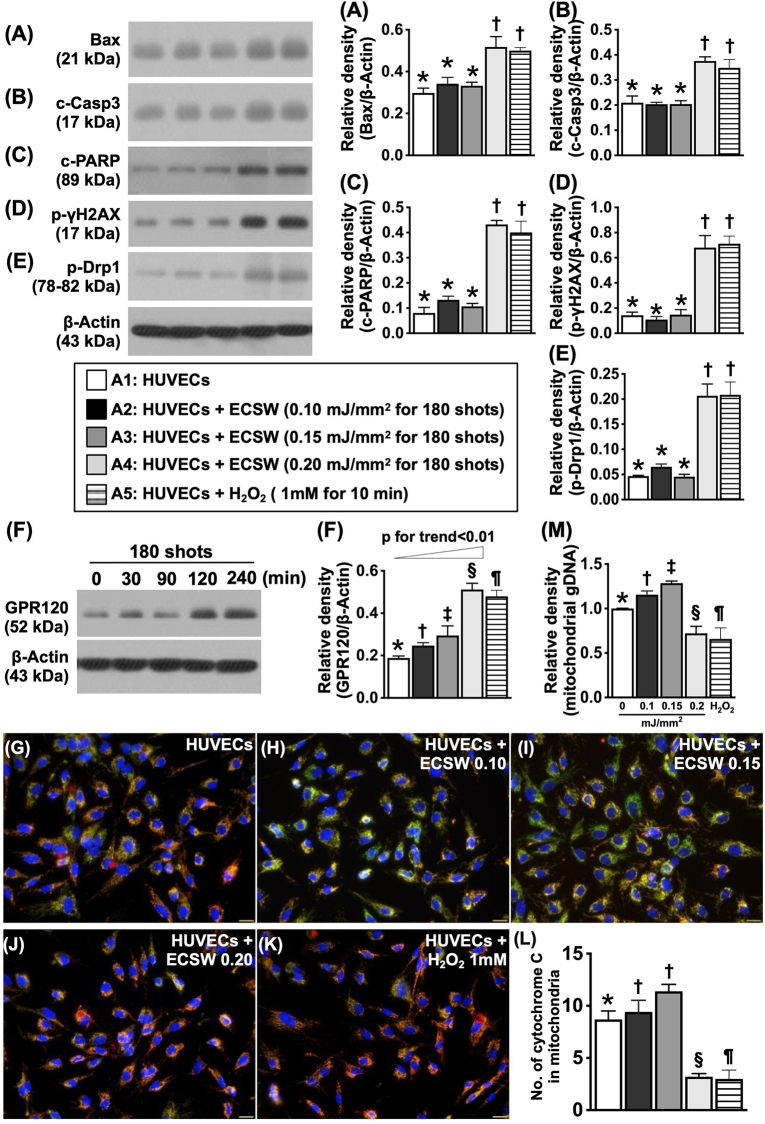
To confirm the time courses and energy-dependence of ECSW on cellular biological responses at 12 h after ECSWT. (A) Protein expression of Bax, * vs. †, *P* < 0.001. (B) Protein expression of cleaved (C) caspase 3 (C-casp3), * vs. †, *P* < 0.001. (C) Protein expression of cleaved PARP, * vs. †, *P* < 0.001. (D) Protein expression of γ-H2AX, * vs. †, *P* < 0.001. (E) Protein expression of phosphorylated (P)-DRP1, * vs. †, *P* < 0.001. (F) Time courses of protein expression of GPR120 undergoing ECSWT (i.e., 0.15 mJ/mm^2^ for 180 shots), * vs. †, *P* < 0.001; † vs. ‡, *P* < 0.05; *P* for trend <0.01. (G–K) Illustrating the immunofluorescent (IF) microscopic finding (400×) for identification of mitochondrial cytochrome C (red-green color, i.e., the merge of cytochrome C staining [green color] and heart shock protein 60 [Hsp60] stain of red color for identification of mitochondria]). Scale bar in right lower corner represents 20 µm. (L) Analytical result of number of cytochrome c in mitochondria, * vs. other groups with different symbols (†, ‡, §, ¶), *P* < 0.0001. (M) qPCR demonstrated relative mitochondrial DNA expression in HUVEC with different energy doses of ECSW and H_2_O_2_ treatment, * vs. other groups with different symbols (†, ‡, §, ¶), *P* < 0.0001. gDNA indicated genomic DNA. All statistical analyses were performed by one-way ANOVA, followed by Bonferroni multiple comparison post hoc test (*N* = 3–5 for each group). Symbols (*, †, ‡, §, ¶) indicate significance (At 0.05 level). ECSWT, extracorporeal shock wave treatment; HUVECs, human umbilical vein endothelial cells; A1, HUVECs; A2, HUVECs + ECSW (0.10 mJ/mm^2^ for 180 shots); A3, HUVECs + ECSW (0.15 mJ/mm^2^ for 180 shots); A4, HUVECs + ECSW (0.20 mJ/mm^2^ for 180 shots); A5, HUVECs + H_2_O_2_ (1 mM treated for 10 min).

Next, to determine whether ECSWT increased the protein expression of GPR120 in a time-dependent manner, HUVECs were collected at different time points (0, 30, 90, 120, and 240 min) after shock wave treatment. The energy level and frequency were set to 0.15 mJ/mm^2^ and 180 impulses, respectively. Fig. 1B shows that the maximal protein expression of GPR120 was identified at 120 min.

### Confirming the energy dependence and time course of the effects of ECSWT on cellular biological responses

In this in vitro study, the HUVECs were categorized as follows: A1 (HUVECs), A2 (HUVECs + ECSWT [0.10 mJ/mm^2^ for 180 shots]), A3 (HUVECs + ECSWT [0.15 mJ/mm^2^ for 180 shots]), A4 (HUVECs + ECSWT [0.20 mJ/mm^2^ for 180 shots]), and A5 (HUVECs + H_2_O_2_ [1 mM treated for 10 min]). Afterward, the cells were incubated for 12 h and then collected for individual study. The protein expression levels of Bax, cleaved caspase 3, and cleaved PARP, three indicators of apoptosis; the protein expression level of γ-H2AX, an index of DNA damage; and the protein expression level of phosphorylated (p)-DRP1, an indicator of mitochondrial damage, were significantly greater in A4 and A5 than in A1 to A3, but they did not differ between A4 and A5 or among A1, A2, and A3 (Fig. 2).

When the time-dependent effect of this energy dose of ECSWT on protein expression was examined, we once again found that 120 min after ECSWT was the earliest time point for maximal protein expression of GPR120 in HUVECs.

In addition, the IF microscopic findings demonstrated that the number of mitochondrial cytochrome C molecules, an indicator of mitochondrial integrity, significantly and progressively increased from A1 to A3 and was markedly reduced in A4 and A5. These findings statistically confirmed that the safe and optimal energy dose of ECSWT for initiating the best cellular biological effect could be 0.15 mJ/mm^2^. On the other hand, the application of a higher energy level of ECSWT up to 0.20 mJ/mm^2^ could result in an effect similar to H_2_O_2_-induced cell damage.

When we examined the gene expression of mitochondrial DNA, we found that the expression of this parameter was similar to that of mitochondrial cytochrome C among the groups.

### ECSWT activated the GPR120-mediated signaling pathway against inflammatory responses

**Figure 3.**
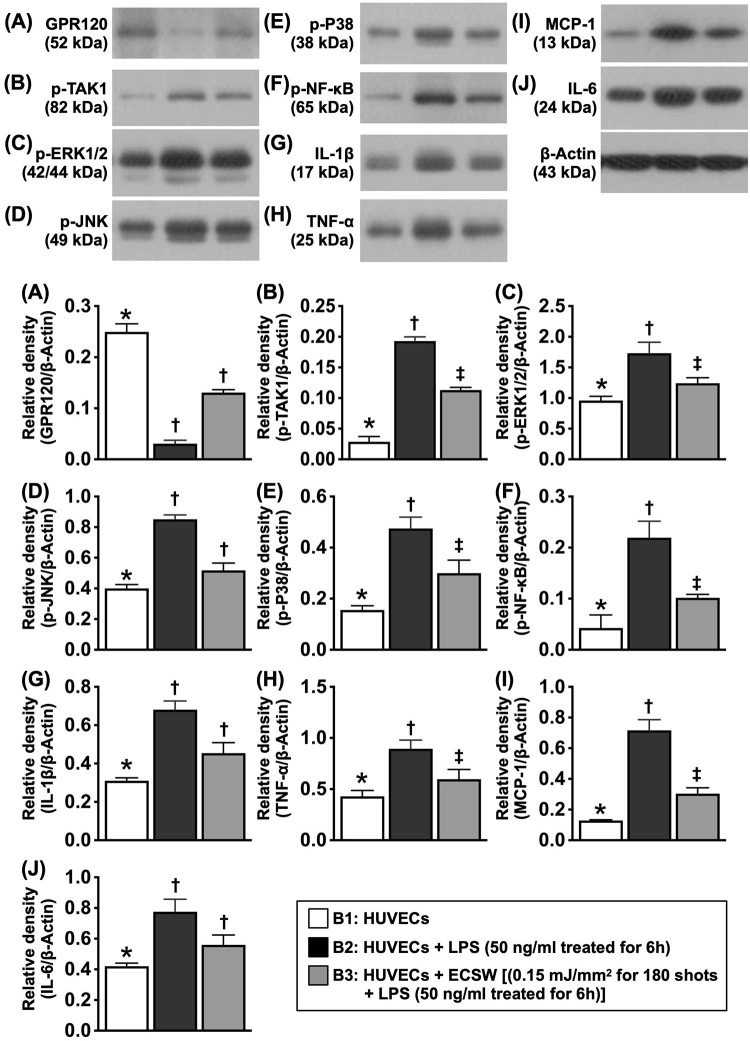
ECSWT activated GPR120-mediated signaling pathway against inflammatory responses. (A) Protein expression of GPR120, * vs. other groups with different symbols (†, ‡), *P* < 0.001. (B) Protein expression of phosphorylated (P) transforming growth factor (TGF)-β active kinase 1 (P-tak1), * vs. other groups with different symbols (†, ‡), *P* < 0.001. (C) Protein expression of p-ERK1/2, * vs. other groups with different symbols (†, ‡), *P* < 0.001. (D) Protein expression of p-JNK, * vs. other groups with different symbols (†, ‡), *P* < 0.0001. (E) Protein expression of P-p38, * vs. other groups with different symbols (†, ‡), *P* < 0.001. (F) Protein expression of P-nuclear factor (p-NF)-κB, * vs. other groups with different symbols (†, ‡), *P* < 0.001. (G) Protein expression of interleukin (IL)-1β, vs. other groups with different symbols (†, ‡), *P* < 0.001. (H) Protein expression of tumor necrosis factor (TNF)-α, vs. other groups with different symbols (†, ‡), *P* < 0.001. (I) Protein expression of monocyte chemoattractant protein (MCP)-1, * vs. other groups with different symbols (†, ‡), *P* < 0.0001. (J) Protein expression of IL-6, * vs. other groups with different symbols (†, ‡), *P* < 0.001. All statistical analyses were performed by one-way ANOVA, followed by Bonferroni multiple comparison post hoc test (*N* =3 for each group). Symbols (*, †, ‡) indicate significance (at 0.05 level). ECSW, extracorporeal shock wave; HUVECs, human umbilical vein endothelial cells; B1, HUVECs; B2, HUVECs + LPS (50 Ng/mL treated for 6 h); B3, HUVECs + ECSW ([0.15 mJ/mm^2^ for 180 shots] + LPS [50 Ng/mL treated for 6 h]).

GPR120 activation with ω-3 FAS triggers the coupling of β-arrestin-2 and inhibits the release of TGF-β active kinase 1 (TAK1), resulting in inactivation of TAK1 and abolishment of inflammatory cascades as well as the signaling processes of NF-κB and mitogen-activated protecting kinases (MAPKs). To determine whether GPR120 is activated by ECSWT, resulting in attenuation of the inflammatory reaction, the HUVECs were categorized into B1 (HUVECs cultured in medium), B2 (HUVECs + LPS [50 ng/mL treated for 6 h, followed by washing and culture for 12 h [i.e., total culture time was 18 h]), and B3 (HUVECs + LPS [50 ng/mL treated for 6 h]) and then washed, followed by ECSWT (0.15 mJ/mm^2^) for 180 shots and then cultured again for another 12 h (i.e., total culture time was 18 h). The cells were finally collected for Western blot analysis (Fig. 3).

Compared with that in B1 (i.e., the control group), the protein level of GPR120 was significantly lower in B2, which was significantly reversed in B3 (i.e., the ECSW-treated group). These findings suggest that inflammation is suppressed, whereas ECSWT upregulates the expression of GPR120, which is known as an anti-inflammatory mediator.

In addition, the protein expression of TAK1, a member of the MAPK kinase family that interacts with TABs to form a TAK1-TAB complex, which is a central signalosome in inflammatory responses, was significantly greater in B2 than in B1, which was substantially reversed in B3, indicating that TAK1 could play an essential role in the initiation and propagation of inflammation that could be notably suppressed by ECSWT. Furthermore, the protein expression levels of p-ERK1/2, p-JNK, and p-p38, three members of the MAPK family, were identical to that of TAK1. Moreover, the protein expression levels of p-NF-κB, IL-1β, TNF-α, MCP-1, and IL-6, five indices of pro-inflammatory cytokines, also displayed a pattern identical to that of TAK1. Taken together, these findings suggested that ECSWT switched on the GPR120-mediated signaling pathway against inflammatory responses.

### ECSWT-induced anti-inflammatory effects were abolished by the GPR120 antagonist and knockdown of GPR120

**Figure 4.**
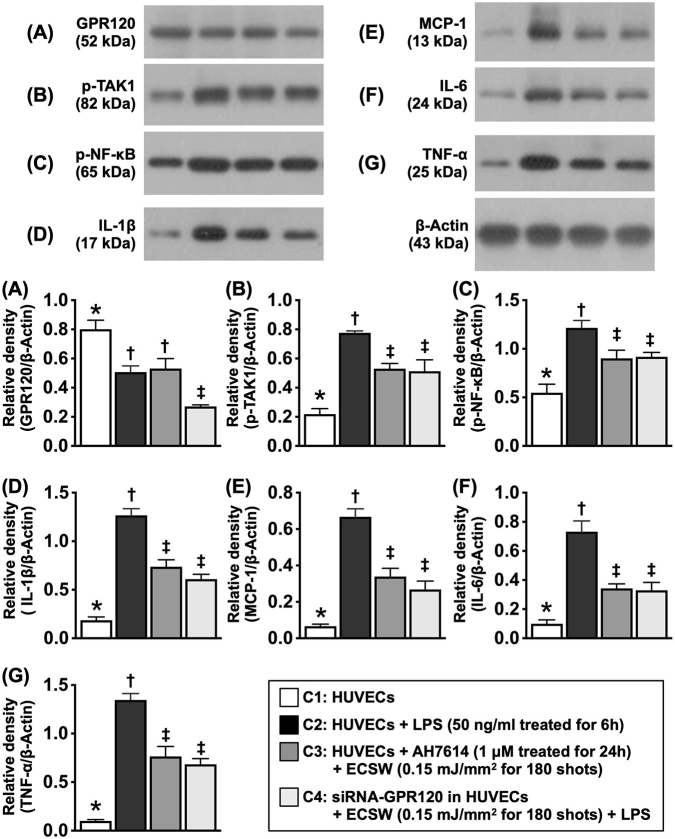
ECSWT-induced anti-inflammatory effect abolished by GPR120 antagonist and knockdown of GPR120. (A) Protein expression of GPR120, * vs. other groups with different symbols (†, ‡), *P* < 0.001. (B) Protein expression of phosphorylated (P)-tak1, * vs. other groups with different symbols (†, ‡), *P* < 0.001. (C) p-NF-κB, * vs. other groups with different symbols (†, ‡), *P* < 0.001. (D) Protein expression of IL-1β, * vs. other groups with different symbols (†, ‡), *P* < 0.001. (E) Protein expression of MCP-1, * vs. other groups with different symbols (†, ‡), *P* < 0.001. (F) Protein expression of IL-6, * vs. other groups with different symbols (†, ‡), *P* < 0.001. (G) Protein expression of TNF-α, * vs. other groups with different symbols (†, ‡), *P* < 0.001. All statistical analyses were performed by one-way ANOVA, followed by Bonferroni multiple comparison post hoc test (*N* = 3 for each group). Symbols (*, †, ‡) indicate significance (at 0.05 level). ECSW, extracorporeal shock wave; HUVECs, human umbilical vein endothelial cells; LPS, lipopolysaccharide; C1, HUVECs; C2, HUVECs + LPS (50 Ng/mL treated for 6 h); C3, HUVECs + AH7614 (1 μM treated for 24 h) + ECSW (0.15 mJ/mm^2^ for 180 shots) + LPS; C4, siRNA-GPR120 in HUVECs + ECSW (0.15 mJ/mm^2^ for 180 shots) + LPS.

The chemical compound AH7614 is well recognized as an inhibitor of the GPR120-mediated signaling pathway in several cell types. To delineate the role of GPR120 in the anti-inflammatory effects of ECSWT, the levels of phosphorylated TAK1 and NF-κB were determined in AH7614-pretreated HUVECs after ECSWT. In addition, small interfering-GPR120 (si-GPR120) was used to silence GPR120 expression to determine the important role of GPR120 after ECSWT. In this in vitro study, the cells were categorized as C1 (HUVECs cultured in medium), C2 (HUVECs + LPS [50 ng/mL] treated for 6 h, followed by washing and culture again for 12 h [i.e., the total culture time was 18 h]), C3 (HUVECs + LPS [50 ng/mL] treated for 6 h and washed, followed by ECSWT [0.15 mJ/mm^2^] for 180 shots, AH7614 [1 μM] treated for 12 h, and cultured again for 12 h [i.e., total culture for 18 h]), and C4 (siRNA-GPR120 in HUVECs + LPS [50 ng/mL] treated for 6 h and washed, followed by ECSWT [0.15 mJ/mm^2^] for 180 shots, and then cultured for 12 h [i.e., the total culture time was 24 h]) (Fig. 4).

The results demonstrated that the protein expression of GPR120 was significantly lower in C2 than in C1, indicating that inflammation markedly suppressed GPR120 expression. In contrast, when the GPR120 gene was silenced or the GPR120 inhibitor AH7614 was used, the protein expression of GPR120 was significantly suppressed even under ECSWT. On the other hand, the protein expression levels of p-TAK1, p-NF-κB, IL-1β, MCP-1, IL-6, and TNF-α, six indicators of inflammation, were opposite those of GPR120 among the groups. Our findings suggested that the anti-inflammatory effect of ECSWT-upregulated GPR120 was reversed by a specific inhibitor or by silencing the GPR120 gene.

### Protein expression associated with inflammation, anti-inflammation, and cell-stress signaling in response to different management strategies in the myoblast cell line C2C12

**Figure 5.**
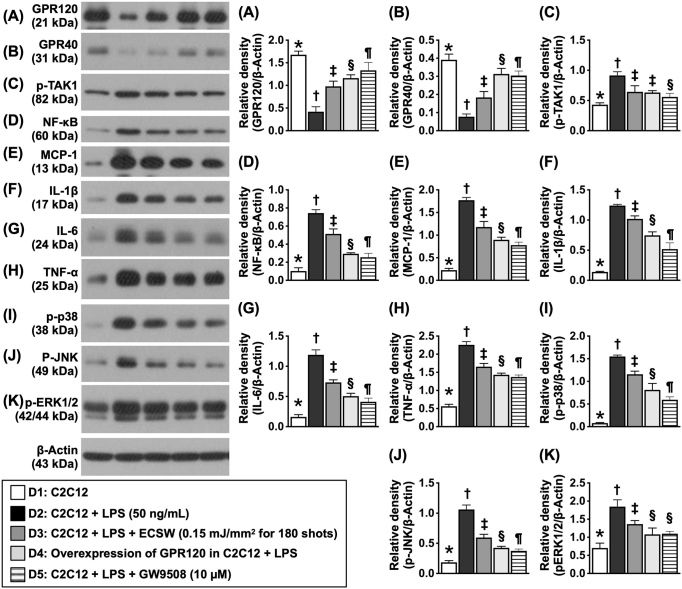
Protein expressions of inflammation, anti-inflammation, and cell-stress signaling in response to different strategic management in myoblast cell line of C2C12 cells. (A) Protein expression of GPR120, * vs. other groups with different symbols (†, ‡, §, ¶), *P* < 0.0001. (B) Protein expression of GPR40, * vs. other groups with different symbols (†, ‡, §, ¶), *P* < 0.0001. (C) Protein expression of phosphorylated (P)-tak1, * vs. other groups with different symbols (†, ‡, §), *P* < 0.0001. (D) Protein expression of P-unclear factor (NF)-κB, * vs. other groups with different symbols (†, ‡, §, ¶), *P* < 0.0001. (E) Protein expression of monocyte chemoattractant protein (MCP)-1, * vs. other groups with different symbols (†, ‡, §, ¶), *P* < 0.0001. (F) Protein expression of interleukin (IL)-1β, * vs. other groups with different symbols (†, ‡, §, ¶), *P* < 0.0001. (G) Protein expression of IL-6, * vs. other groups with different symbols (†, ‡, §, ¶), *P* < 0.0001. (H) Protein expression of tumor necrosis factor (TNF)-α, * vs. other groups with different symbols (†, ‡, §, ¶), *P* < 0.0001. (I) Protein expression of p-P38, * vs. other groups with different symbols (†, ‡, §, ¶), *P* < 0.0001. (J) Protein expression of p-JNK, * vs. other groups with different symbols (†, ‡, §, ¶), *P* < 0.0001. (K) Protein expression of ERK1/2, * vs. other groups with different symbols (†, ‡, §), *P* < 0.0001. All statistical analyses were performed by one-way ANOVA, followed by Bonferroni multiple comparison post hoc test (*N* =3 for each group). Symbols (*, †, ‡, §, ¶) indicate significance (at 0.05 level). Cell groups: D1, C2C12; D2, C2C12 + LPS (50 Ng/mL); D3, C2C12 + L + LPS (50 Ng/mL) + ECSWT (0.15 mJ/mm^2^) for 180 shots; D4, overexpression of GPR in C2C12 + LPS (50 Ng/mL); D5, C2C12 + LPS (50 ng/mL) + GW9508 (10 µm).

In this in vitro study, the C2C12 cell line was categorized as D1 (C2C12), D2 (C2C12 + LPS [50 ng/mL] treated for 6 h, and washed and cultured again for 12 h [i.e., total culture time was 18 h]), D3 (C2C12 + L + LPS [50 ng/mL] treated for 6 h and washed, followed by ECSWT [0.15 mJ/mm^2^] for 180 shots and cultured again for 12 h [i.e., total culture for 18 h]), D4 (overexpression of GPR in C2C12 + LPS [50 ng/mL] treated for 6 h and washed and cultured again for 12 h [i.e., total culture for 18 h]), and D5 (C2C12 + LPS [50 ng/mL] treated for 6 h and washed + GW9508 [10 µM, i.e., an agonist of GPR] treated for 12 h and cultured again for 12 h [i.e., total culture for 18 h)] (Fig. 5).

The results revealed that the protein expression levels of GPR120 and GPR40 were highest at D1, lowest at D2 and significantly increased progressively from D3 to D5. On the other hand, the protein expression levels of phosphorylated (p)-TAK1, p-NF-κB, MCP-1, IL-1β, IL-6, and TNF-α, which are six indices of inflammation, exhibited the opposite pattern of GPR120 among the groups. In addition, the protein expression levels of p-P38, p-JNK, and ERK1/2, three members of the MAPK family, which are cell stress biomarkers, also exhibited patterns opposite to those of GPR120 among the groups. Our findings once again proved that GPR120 could be essential for alleviating the inflammatory reaction.

### The time courses of the ratio of INBF in the CLI area

**Figure 6.**
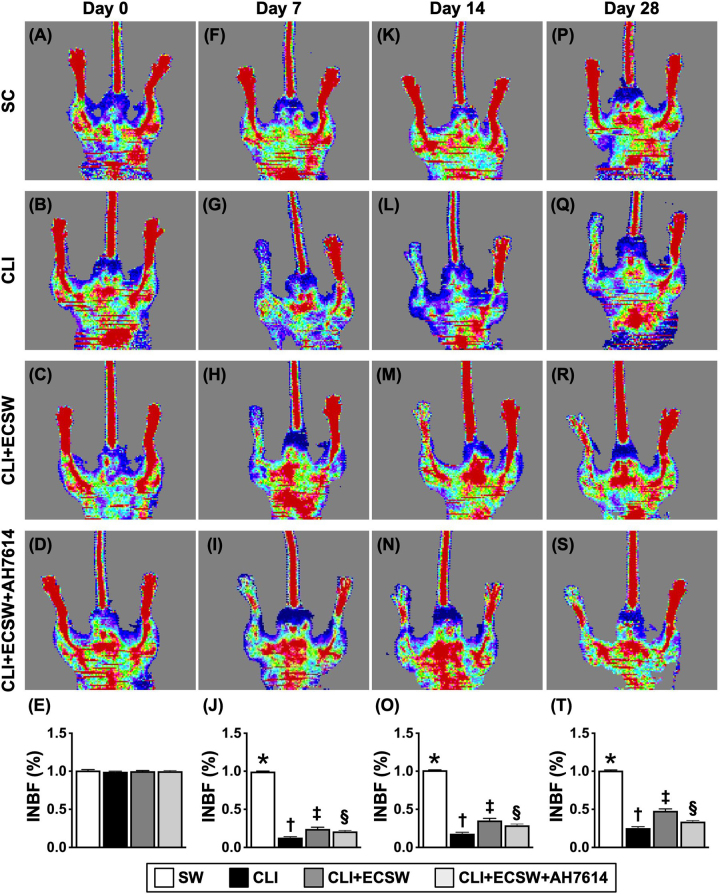
The Time courses of the ratio of ischemia to normal blood flow (INBF) in CLI area. (A–D) Illustrating the laser Doppler for identification of blood flow in right and left inguinal areas at baseline. (E) Analytical result of the ratio of INBF at Day 0, *P* > 0.5. (F–I) Illustrating the laser Doppler for identification of blood flow in right and left inguinal areas by Day 7 after CLI induction. (J) Analytical result of ratio of INBF by Day 7 after CLI induction, * vs. other groups with different symbols (†, ‡, §), *P* < 0.0001. (K–N) Illustrating the laser Doppler for identification of blood flow in right and left inguinal areas by Day 14 after CLI induction. (O) Analytical result of ratio of INBF by Day 14 after CLI induction, * vs. other groups with different symbols (†, ‡, §), *P* < 0.0001. (P–S) Illustrating the laser Doppler for identification of blood flow in right and left inguinal areas by Day 28 after CLI induction. (T) Analytical result of ratio of INBF by Day 28 after CLI induction, * vs. other groups with different symbols (†, ‡, §), *P* < 0.0001. Note that AH7614 is an Inhibitor of GPR120. All statistical analyses were performed by one-way ANOVA, followed by Bonferroni multiple comparison post hoc test (*N* = 8–10 for each group). Symbols (*, †, ‡, §) indicate significance (at 0.05 level). SC, sham-operated control; CLI, critical limb ischemia; ECSWT, extracorporeal shock wave therapy.

By Day 0, the ratio of INBF did not differ among Groups 1 (SC), 2 (CLI), 3 (CLI + ECSW), and 4 (CLI + ECSW + AH7614). However, by Day 3 after CLI induction, the ratio of INBF was significantly greater in Group 1 than in Groups 2–4, but this parameter did not differ among the latter three groups. On the other hand, by Days 7, 14, and 28 after CLI induction, this parameter was highest in Group 1, lowest in Group 2, and significantly greater in Group 3 than in Group 4, indicating that the ability of ECSWT to restore blood flow in the CLI area was markedly attenuated by the GPR120 inhibitor AH7614. These findings confirmed that ECSWT restored blood flow in the ischemic area (Fig. 6).

### Impact of ECSWT on enhancing angiogenesis biomarkers in the ischemic quadriceps area by Day 28 after CLI induction

**Figure 7.**
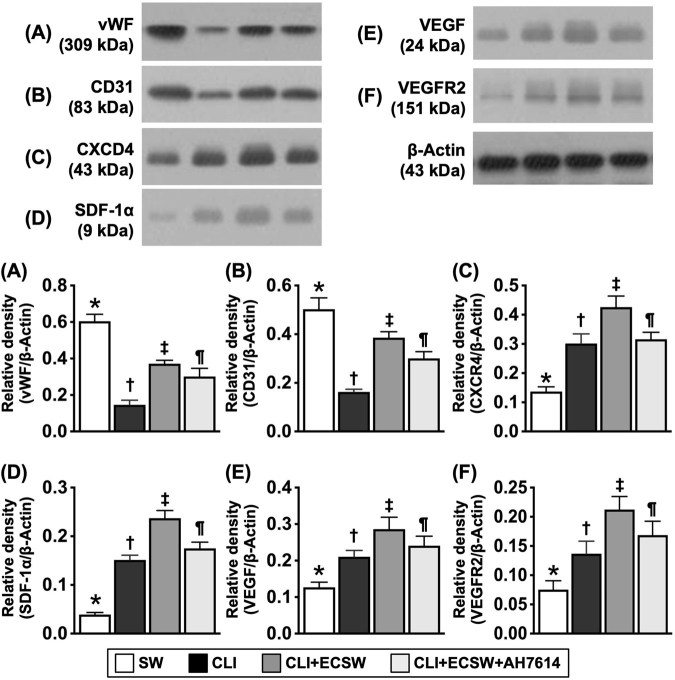
Impact of ECSWT on enhancing the angiogenesis in ischemic quadriceps area by Day 28 after CLI induction. (A) Protein expression of von Willebrand factor (VwF), * vs. other groups with different symbols (†, ‡, §), *P* < 0.0001. (B) Protein expression of CD31, * vs. other groups with different symbols (†, ‡, §), *P* < 0.0001. (C) Protein expression of CXCR4, * vs. other groups with different symbols (†, ‡, §), *P* < 0.0001. (D) Protein expression of stromal cell-derived factor (SDF)-1α, * vs. other groups with different symbols (†, ‡, §), *P* < 0.0001. (E) Protein expression of vascular endothelial growth factor (VEGF), * vs. other groups with different symbols (†, ‡, §), *P* < 0.0001. (F) Protein expression of vascular endothelial growth factor receptor 2 (VEGFR2), * vs. other groups with different symbols (†, ‡, §), *P* < 0.0001. Note that the tissue sample for Western blot analysis was the harvested from quadriceps muscle of CLI Area. All statistical analyses were performed by one-way ANOVA, followed by Bonferroni multiple comparison post hoc test (*N* = 6 for each group). Symbols (*, †, ‡, §) indicate significance (at 0.05 level). SC, sham-operated control; CLI, critical limb ischemia; ECSWT, extracorporeal shock wave therapy.

To evaluate the therapeutic impact of ECSWT on enhancing angiogenesis factors in the ischemic area, the quadriceps muscle was harvested for Western blot analysis. The results revealed that the protein expression levels of vWF and CD31, two indicators of integrity of endothelial cell/angiogenesis markers, were highest in Group 1, lowest in Group 2, and significantly higher in Group 3 than in Group 4. In addition, the protein expression levels of CXCR4, SDF-1, VEGF, and VEGFR2, four indicators of angiogenesis factors, significantly and progressively increased from Groups 1 to 3, followed by a decrease in Group 4, indicating an intrinsic response to ischemic stimulation. However, these four parameters were significantly reversed in Group 4 compared with those in Group 3. These findings confirmed that ECSWT increased angiogenesis through the generation of GPR120, which was attenuated by the GPR120 inhibitor AH7614 (Fig. 7).

### Impact of ECSWT on attenuating oxidative stress and inflammation biomarkers in the ischemic quadriceps area by Day 28 after CLI induction

**Figure 8.**
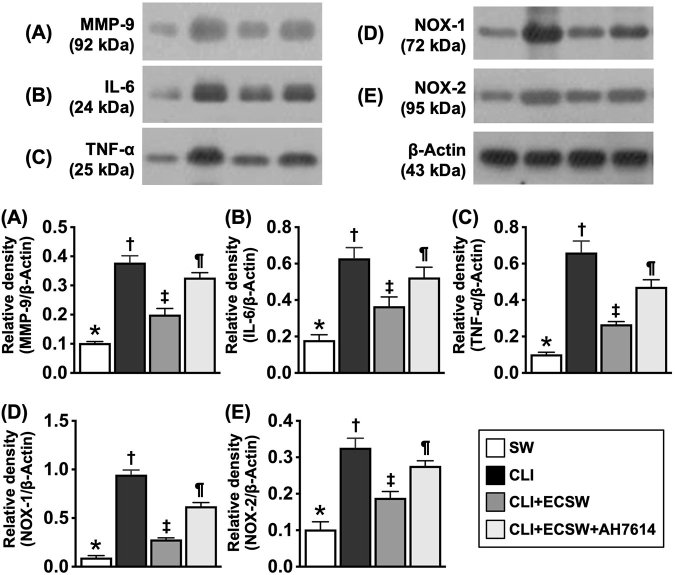
ECSWT attenuated oxidative stress and inflammation in ischemic quadriceps by Day 28 after CLI induction. (A) Protein expression of matrix metalloproteinase (MMP)-9, * vs. other groups with different symbols (†, ‡, §), *P* < 0.0001. (B) Protein expression of interleukin (IL)-6, * vs. other groups with different symbols (†, ‡, §), *P* < 0.0001. (C) Protein expression of tumor necrosis factor (TNF)-α, * vs. other groups with different symbols (†, ‡, §), *P* < 0.0001. (D) Protein expression of NOX-1, * vs. other groups with different symbols (†, ‡, §), *P* < 0.0001. (E) Protein expression of NOX-2, * vs. other groups with different symbols (†, ‡, §), *P* < 0.0001. Note that the tissue sample for Western blot analysis was harvested from quadriceps muscle of CLI area. All statistical analyses were performed by one-way ANOVA, followed by Bonferroni multiple comparison post hoc test (*N* = 6 for each group). Symbols (*, †, ‡, §) indicate significance (at 0.05 level). SC, sham-operated control; CLI, critical limb ischemia; ECSWT, extracorporeal shock wave therapy.

To verify whether ECSWT alleviated oxidative stress and inflammation, Western blot was conducted again for the in vivo study. The results revealed that the protein expression levels of MMP-9, IL-6, and TNF-α, three indicators of inflammation, and the protein expression levels of NOX-1 and NOX-2, two indicators of oxidative stress, were lowest in Group 1, highest in Group 2, and significantly higher in Group 4 than in Group 3. These findings support the mechanism by which ECSWT alleviates inflammation and oxidative stress mainly through GPR120 expression (Fig. 8).

## Cellular expression associated with inflammation and DNA damage by day 28 after CLI induction

In addition to assessing the molecular levels, we wanted to evaluate whether the cellular levels of inflammation and DNA damage were also alleviated by ECSWT.

**Figure 9.**
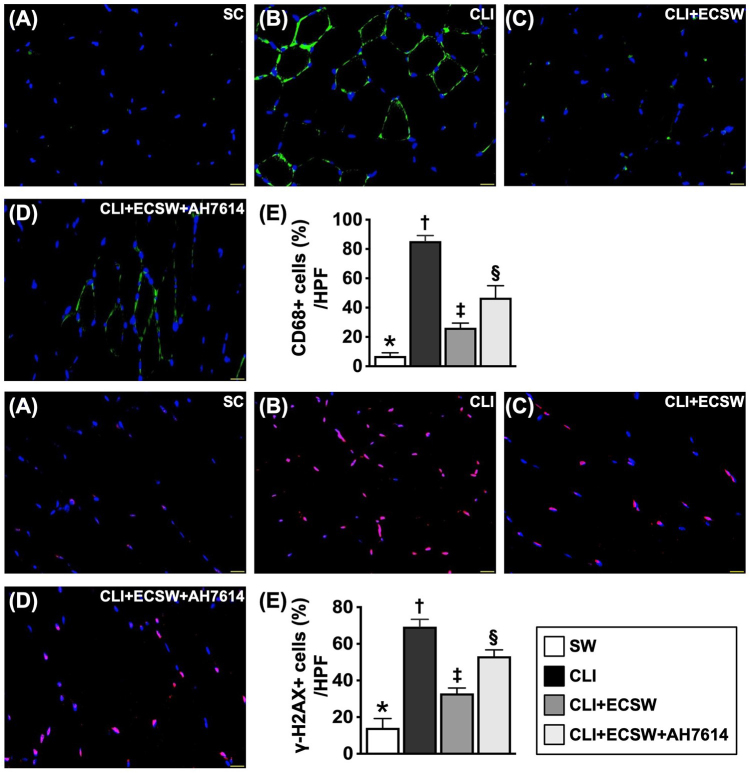
ECSWT alleviated cellular expressions of inflammation and DNA damage by Day 28 after CLI induction. (A–D) Illustrating the immunofluorescent (IF) microscopic finding (400×) for identification of cellular expression of CD68 (green color). (E) Analytical result of cellular expression (%) of CD68, * vs. other groups with different symbols (†, ‡, §), *P* < 0.0001. (F–I) Illustrating the IF microscopic finding (400×) for identification of cellular expression of γ-H2AX (pink color). (J) Analytical result of cellular expression (%) of γ-H2AX, * vs. other groups with different symbols (†, ‡, §), *P* < 0.0001. Scale bar in right lower corner represents 20 µm. Note that the tissue sample for IF stain was harvested from quadriceps muscle of CLI area. All statistical analyses were performed by one-way ANOVA, followed by Bonferroni multiple comparison post hoc test (*N* = 6 for each group). Symbols (*, †, ‡, §) indicate significance (at 0.05 level). SC, sham-operated control; CLI, critical limb ischemia; ECSWT, extracorporeal shock wave therapy.

The IF microscopic findings demonstrated that the cellular expression of CD68, an index of inflammation, and the cellular expression of γ-H2AX, an indicator of DNA damage, were lowest in Group 1, highest in Group 2, and significantly greater in Group 4 than in Group 3 (Fig. 9).

### Vessel density and cellular expression of GPR120 in ischemic quadriceps muscle 28 days after CLI induction

**Figure 10.**
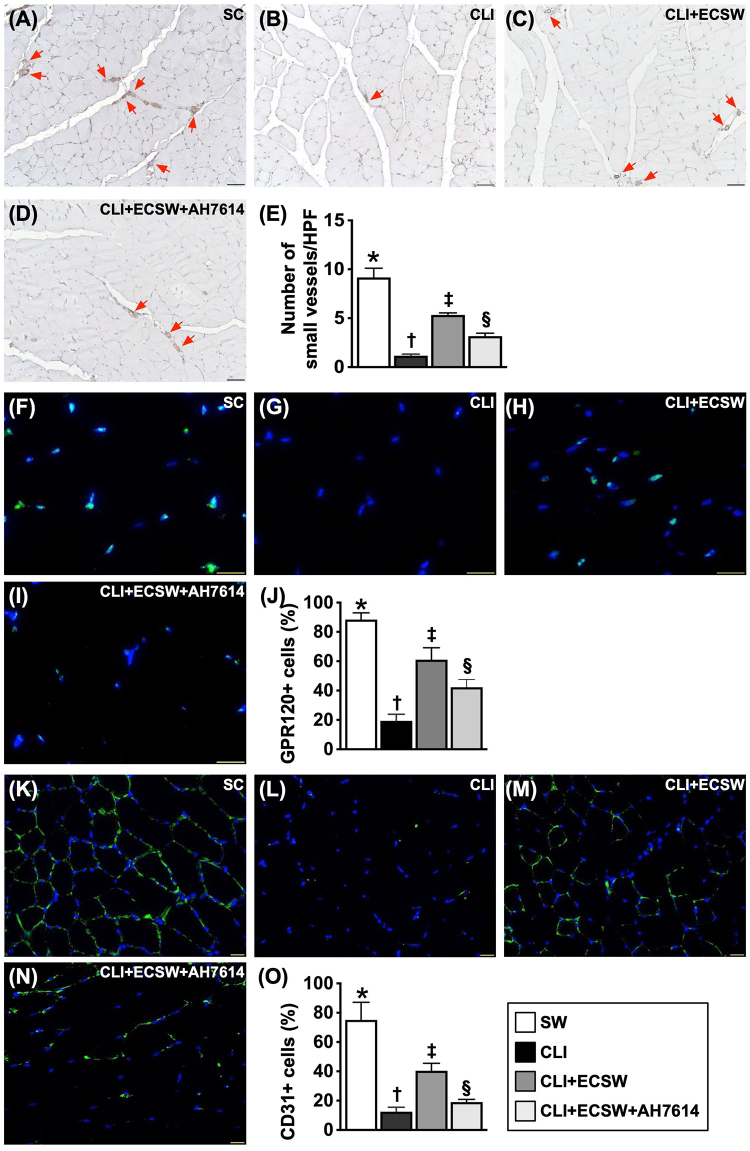
Vessel density and cellular expression of GPR120 in ischemic quadriceps muscle by Day 28 after CLI Induction. (A–D) Illustrating the immunohistochemical (IHC) microscopic finding (100×) of alpha-smooth actin (α-SMA) stain for identification of small vessel density (gray color) (red arrows). (E) Analytical result of number of small vessels (i.e., defined as diameter ≤25 µm), * vs. other groups with different symbols (†, ‡, §), *P* < 0.0001. (F–I) Illustrating the IF microscopic finding (400×) for identification of cellular expression of GPR120 (green color). (J) Analytical result of number of GPR120 + cells, * vs. other groups with different symbols (†, ‡, §), *P* < 0.0001. (K–N) Illustrating the IF microscopic finding (400×) for identification of cellular expression of CD31. (O) Analytical result of number of CD31 + cells, * vs. other groups with different symbols (†, ‡, §), *P* < 0.0001. Scale bar in right lower corner represents 20 µm. All statistical analyses were performed by one-way ANOVA, followed by Bonferroni multiple comparison post hoc test (*N* = 6 for each group). Symbols (*, †, ‡, §) indicate significance (at 0.05 level). SC, sham-operated control; CLI, critical limb ischemia; ECSWT, extracorporeal shock wave therapy.

Finally, we utilized an IHC microscope to verify the small vessel density in the CLI area, and the results revealed that the number of small vessels, an indicator of angiogenesis/neovascularization, was highest in Group 1, lowest in Group 2 and significantly greater in Group 3 than in Group 4. In addition, the cellular expression of GPR120 in the quadriceps muscle of the CLI area exhibited a similar vessel density among the groups (Fig. 10).

## Discussion

This study, which investigated the impact of ECSWT therapy on salvaging CLI in mice, yielded several novel implications. First, this study demonstrated that 0.15 mJ/mm^2^ for 180 shots was the most suitable energy dose for eliciting the biological effects of ECSW. Second, this study revealed that ECSWT augmented the cellular and protein expression of GPR120, which directly participated in anti-inflammatory reactions. Third, ECSWT rescued CLI mainly through increasing angiogenesis and GPR120-mediated anti-inflammatory effects and lowering oxidative stress.

How much energy is to be utilized for the best biological effect is always an interesting matter to be considered^[28]^. To answer this universally inconsistent issue, we conducted a stepwise increase in the energy dose of ECSW in our in vitro study. Intriguingly, we found that while a higher dose (i.e., >0.2 mJ/mm^2^ for 180 shots) of ECSW was harmful to HUVECs, a lower dose (i.e., 0.10 mJ/mm^2^ for 180 shots or less) had an inadequate biological effect. An essential finding in the present study was that the energy dose of 0.15 mJ/mm^2^ for 180 shots was the optimal dose of ECSW energy for initiating the best biological effect. Intriguingly, our previous study^[28]^ demonstrated that 0.12–0.14 mJ/mm^2^ was a suitable ECSW for initiating biomarkers of cell survival signaling. In this way, our findings from the present study, in addition to being comparable with the findings of our previous study,^[28]^ were referenced as a suitable energy dose for our in vivo study.

Abundant data have shown that increased angiogenesis is one of the important biological effects of ECSWT^[8,10,13,14,29]^. Interestingly, our previous study demonstrated that ECSWT significantly enhanced angiogenesis mainly through VEGFR2 activation and recycling^[14]^, indicating that this finding verified the accurate mechanism by which ECSWT improved ischemic events in various ischemic organs^[8,10,13,14,29]^. A principle finding in the present study was that the protein expression of VEGFR in the ischemic quadriceps muscle was markedly enhanced after ECSWT. In addition, the levels of angiogenesis biomarkers were substantially increased in CLI patients after ECSWT. Our findings, in addition to being consistent with the findings of previous studies^[8,10,13,14,29]^, explained, at least in part, why ECSWT could improve blood flow in the CLI area and outcomes.

**Figure 11.**
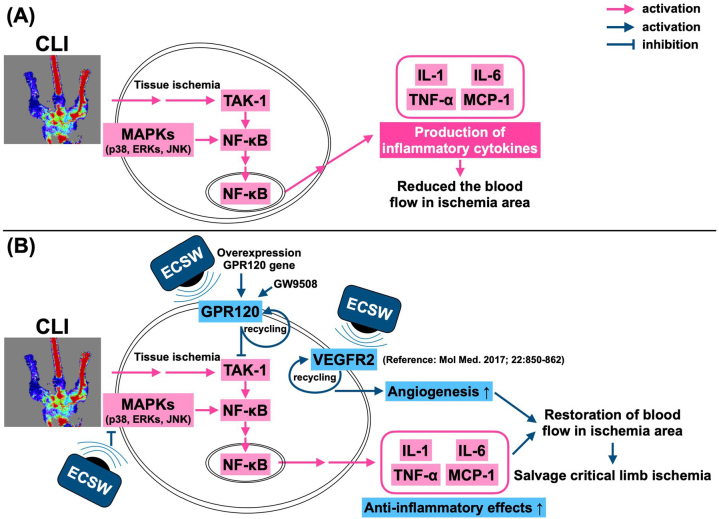
Schematically illustrated the underlying mechanism of ECSWT on upregulation of GPR120 and angiogenesis for salvaging the CLI in rodent. ECSWT, extracorporeal shock wave therapy; TAK1, TGF-β active kinase 1; GPR120, protein-coupled receptor 120; CLI, critical limb ischemia; NF-κB, nuclear factor κB; VEGF, vascular endothelial growth factor; IL, interleukin; TNF, tumor necrosis factor; MCP-1, monocyte chemoattractant protein-1.

Inflammation initiates oxidative stress, mitochondrial damage, delayed wound healing, endothelial and organ dysfunction, and cellular apoptosis and death^[9–12,25,29]^, as well as arterial atherosclerosis and occlusion^[30]^. In addition, many studies have demonstrated that ECSWT has a strong anti-inflammatory effect^[11,12,25,31,32]^. However, the exact mechanism by which ECSWT attenuates the inflammatory reaction remains unclear. The most important finding in the present study was that, compared with those in the SC group, the levels of inflammatory mediators were markedly increased in the CLI groups. However, these inflammatory mediators were markedly suppressed in CLI animals after receiving ECSWT. Interestingly, these parameters were once again upregulated in CLI animals treated with AH7614 (a specific inhibitor of GPR120), even after ECSWT, suggesting that GPR120 (an ω-3 FAS receptor) has a potent anti-inflammatory effect on ECSWT (Fig. 11).

### Study limitations

Our study has several limitations. First, although we utilized a molecular tool for silencing and overexpressing the GPR120 gene in vitro and chemical compounds AH7614 (i.e., an antagonist) and GW9508 (i.e., an agonist) in vivo for the purpose of inhibiting GPR120 activity, without conditional knockdown of GPR120 in the animals, we still cannot completely rule out other anti-inflammatory signaling(s) that mediate the effect of ECSWT in the context of CLI. Second, although the results were attractive and promising, the study period of 28 days should be relatively short; thus, any conclusion of long-term outcomes after ECSWT in CLI animals could not be extrapolated. Third, although overexpression, knockdown, inhibitor, and agonist of GPR were utilized in the present study and the results are promising, without the use of genetic modification experiments, this study could not directly prove that GPR120 has an independent role in improving CLI outcomes.

In conclusion, the results of the present study demonstrated that ECSWT effectively rescued CLI via upregulating GPR120 to prevent inflammation and promote angiogenesis to restore blood flow in the CLI area.

## Data Availability

The datasets of present study can be available from the corresponding author upon request.
